# High Sequence Variability of the *ppE18* Gene of Clinical *Mycobacterium tuberculosis* Complex Strains Potentially Impacts Effectivity of Vaccine Candidate M72/AS01E

**DOI:** 10.1371/journal.pone.0152200

**Published:** 2016-03-24

**Authors:** Susanne Homolka, Tanja Ubben, Stefan Niemann

**Affiliations:** 1 Molecular and Experimental Mycobacteriology, Research Center Borstel, Schleswig–Holstein, Germany; 2 German Centre for Infection Research (DZIF), Partner Site Borstel, Schleswig–Holstein, Germany; University of Minnesota, UNITED STATES

## Abstract

The development of an effective vaccine is urgently needed to fight tuberculosis (TB) which is still the leading cause of death from a single infectious agent worldwide. One of the promising vaccine candidates M72/AS01E consists of two proteins subunits PepA and PPE18 coded by Rv0125 and Rv1196. However, preliminary data indicate a high level of sequence variability among clinical *Mycobacterium tuberculosis* complex (MTBC) strains that might have an impact on the vaccine efficacy. To further investigate this finding, we determined *ppE18* sequence variability in a well-characterized reference collection of 71 MTBC strains from 23 phylogenetic lineages representing the global MTBC diversity. In total, 100 sequence variations consisting of 96 single nucleotide polymorphisms (SNPs), three insertions and one deletion were detected resulting in 141 variable positions distributed over the entire gene. The majority of SNPs detected were non-synonymous (n = 68 vs. n = 28 synonymous). Strains from animal adapted lineages, e.g., *M*. *bovis*, showed a significant higher diversity than the human pathogens such as *M*. *tuberculosis* Haarlem. SNP patterns specific for different lineages as well as for deeper branches in the phylogeny could be identified. The results of our study demonstrate a high variability of the *ppE18* gene even in the N-terminal domains that is normally highly conserved in *ppe* genes. As the N-terminal region interacts with TLR2 receptor inducing a protective anti-inflammatory immune response, genetic heterogeneity has a potential impact on the vaccine efficiency, however, this has to be investigated in future studies.

## Introduction

Development of an effective vaccine to fight tuberlulosis (TB) which is still one of the leading infectious diseases worldwide is an ongoing endeavor. In 2014, an estimated 9.0 million people developed TB and 1.5 million died [1]. Infected people have a 10% lifetime risk to get active TB disease and every TB patient can infect up to 10–15 other people/year on average [2]. To break this cycle, a new vaccine is an important component. So far, the only available prevention to develop severe forms of pediatric non-pulmonary TB is the live Bacille Calmette-Guérin (BCG) vaccine from 1921. However, it is virtually not effective against adult pulmonary TB, which is responsible for most of the TB disease burden worldwide[3]. During the last years, ongoing research work resulted in more than 16 promising TB vaccine candidates with different strategies like viral vectored or protein adjuvant booster vaccines as well as priming and therapeutic vaccines [4,5]. One of the in clinical phase II candidates is the M72/AS01E vaccine developed to boost BCG- or *M*. *tuberculosis* induced immune responses [4]. The M72 consist of the modified Mtb72F antigen and the AS01E adjuvant containing different immune stimulants [4,6]. Mtb72F comprises two proteins subunits coded by Rv1196 (*ppE18*, *mtb39a*) and Rv0125 (*pepA*, *mtb32a*) [7]. Although Mtb72F/AS01E showed already encouraging results in diverse animal models e.g. guinea pigs and cynomolgus monkeys [8,9] effectiveness of the vaccine candidates is potentially challenged by DNA polymorphisms in the *ppE18* gene [10,11].

TB is caused by members of the *M*. *tuberculosis* complex (MTBC) comprising twelve different species (*M*. *tuberculosis*, *M*. *africanum*, *M*. *canettii*, *M*. *bovis*, *M*. *caprae*, *M*. *microti*, *M*. *pinipedii*, *M*. *origys*, *M*. *mungi*, *M*. *suricattae*, the dassie bacillus and the chim-panzee bacillus) which can be further distinguished in at least two major phylogenetic groups (clade 1 and clade 2) and more than 20 phylogenetic lineages/genotypes [12,13]. In several studies, it was shown that the genetic variability of MTBC strains impacts the outcome of infection [14,15], the transmission probability [16,17], the success of spreading of resistant strains [18,19], virulence mechanism [20,21] and the performance of TB vaccines [10,22,23]. Especially with regard to the development of new vaccines, the investigation of the genetic diversity of target genes is indispensable for a maximum of vaccine efficacy. Hebert et al. investigated the genomic diversity of the *ppE18* gene among 225 clinical isolates from two different geographical regions [10]. Single nucleotide polymorphisms (SNPs) as well as insertions (INS) or deletions (DEL) occurred in more than 20% of all strains investigated. Sequence variations were correlated to the three principal genetic groups 1–3 defined by Sreevatsan et al. [24]. However, a thorough analysis of the sequence variability of the *ppE18* gene among strains representing the global diversity of the MTBC with several distinct phylogenetic groups and lineages/genotypes has not yet been performed.

To address this question, we investigated the sequence diversity of the *ppE18* gene in a well characterized reference collection of 68 clinical MTBC isolates (Table 1) representing 23 phylogenetic lineages as well as the ATCC strains H37Rv (ATCC 27294), *M*. *africanum* (ATCC 25420), and *M*. *bovis* (ATCC 19210).

**Table 1:** Strains of the reference collection.

Sample Name	Species	Lineage/Genotype	Phylogenetic Group
1449/02	*M*. *africanum*	West African 1a	Clade 2
1473/02	*M*. *africanum*	West African 1a	Clade 2
5434/02	*M*. *africanum*	West African 1a	Clade 2
10473/01	*M*. *africanum*	West African 1b	Clade 2
10494/01	*M*. *africanum*	West African 1b	Clade 2
1443/02	*M*. *africanum*	West African 1b	Clade 2
10514/01	*M*. *africanum*	West African 2	Clade 2
10517/01	*M*. *africanum*	West African 2	Clade 2
5468/02	*M*. *africanum*	West African 2	Clade 2
9550/00	*M*. *africanum*	West African 2 ATCC 25420	Clade 2
4258/00	*M*. *bovis*	Bovis	Clade 2
751/01	*M*. *bovis*	Bovis	Clade 2
7540/01	*M*. *bovis*	Bovis	Clade 2
9564/00	*M*. *bovis*	Bovis ATCC 19210	Clade 2
3040/99	*M*. *canettii*	Canettii	Clade 2
3041/99	*M*. *canettii*	Canettii	Clade 2
3151/08	*M*. *canetti*	Canettii	Clade 2
1694/00	*M*. *caprae*	Caprae	Clade 2
8986/99	*M*. *caprae*	Caprae	Clade 2
9577/99	*M*. *caprae*	Caprae	Clade 2
416/01	*M*. *microti*	Llama	Clade 2
8753/00	*M*. *microti*	Llama	Clade 2
1479/99	*M*. *microti*	Vole	Clade 2
7011/02	*M*. *pinipedii*	Seal	Clade 2
7739/01	*M*. *pinipedii*	Seal	Clade 2
12594/02	*M*. *tuberculosis*	Beijing	Clade 1
1500/03	*M*. *tuberculosis*	Beijing	Clade 1
1934/03	*M*. *tuberculosis*	Beijing	Clade 1
3256/02	*M*. *tuberculosis*	Beijing	Clade 1
3329/02	*M*. *tuberculosis*	Beijing	Clade 1
4445/02	*M*. *tuberculosis*	Beijing	Clade 1
1417/02	*M*. *tuberculosis*	Cameroon	Clade 1
5390/02	*M*. *tuberculosis*	Cameroon	Clade 1
5400/02	*M*. *tuberculosis*	Cameroon	Clade 1
2637/02	*M*. *tuberculosis*	Delhi/CAS	Clade 1
7936/01	*M*. *tuberculosis*	Delhi/CAS	Clade 1
9915/01	*M*. *tuberculosis*	Delhi/CAS	Clade 1
1797/03	*M*. *tuberculosis*	EAI	Clade 2
4850/03	*M*. *tuberculosis*	EAI	Clade 2
947/01	*M*. *tuberculosis*	EAI	Clade 2
10469/01	*M*. *tuberculosis*	Ghana	Clade 1
10493/01	*M*. *tuberculosis*	Ghana	Clade 1
2570/02	*M*. *tuberculosis*	Ghana	Clade 1
9679/00	*M*. *tuberculosis*	H37Rv ATCC 27294	Clade 1
2336/02	*M*. *tuberculosis*	Haarlem	Clade 1
4130/02	*M*. *tuberculosis*	Haarlem	Clade 1
9532/03	*M*. *tuberculosis*	Haarlem	Clade 1
7968/03	*M*. *tuberculosis*	LAM	Clade 1
8885/03	*M*. *tuberculosis*	LAM	Clade 1
946/03	*M*. *tuberculosis*	LAM	Clade 1
10459/03	*M*. *tuberculosis*	New-1	Clade 1
12591/02	*M*. *tuberculosis*	New-1	Clade 1
8870/03	*M*. *tuberculosis*	New-1	Clade 1
2151/03	*M*. *tuberculosis*	S-type	Clade 1
2318/06	*M*. *tuberculosis*	S-type	Clade 1
6411/05	*M*. *tuberculosis*	S-type	Clade 1
11313/03	*M*. *tuberculosis*	Tur	Clade 1
10264/03	*M*. *tuberculosis*	Tur	Clade 1
10529/03	*M*. *tuberculosis*	Tur	Clade 1
2169/99	*M*. *tuberculosis*	Uganda I	Clade 1
2201/99	*M*. *tuberculosis*	Uganda I	Clade 1
2333/99	*M*. *tuberculosis*	Uganda I	Clade 1
2176/99	*M*. *tuberculosis*	Uganda II	Clade 1
2191/99	*M*. *tuberculosis*	Uganda II	Clade 1
2253/99	*M*. *tuberculosis*	Uganda II	Clade 1
1657/03	*M*. *tuberculosis*	Ural	Clade 1
2679/03	*M*. *tuberculosis*	Ural	Clade 1
8431/03	*M*. *tuberculosis*	Ural	Clade 1
4412/04	*M*. *tuberculosis*	X-type	Clade 1
8431/05	*M*. *tuberculosis*	X-type	Clade 1
9953/04	*M*. *tuberculosis*	X-type	Clade 1

ATCC: American Type Culture Collection; CAS: Central Asien; EAI: East African Indian; LAM: Latin American Mediterranean; Tur: Turkish

## Materials and Methods

Strains were cultivated on Löwenstein/Jensen media at the National Reference Center for Mycobacteria in Borstel, Germany and DNA extraction was performed according to a standardized protocol [25]. All strains were analyzed by 24-loci-MIRU-VNTR (Mycobacterial Interspersed Repetitive Units-Variable Number of Tandem Repeats), spoligotyping (Spacer Oligonucleotide typing) as well as deletion analysis (region of difference; RD) to confirm phylogenetic classification (details at www.MIRU-VNTRplus.org). Molecular typing data were analyzed with the BioNumerics software (version 7.5, Applied Maths, Sint-Martens-Latem, Belgium) as instructed by the manufacturers.

For DNA sequence analysis of the *ppE18* gene (Rv1196) polymerase chain reaction (PCR) was performed for all strains of the reference collection. Primer Rv1196F (5`ccc gct gcc gat gag gtg t 3`) was located 223bp upstream of the *ppE18* gene, the reverse primer Rv1196R (5`gag acc gcc gat cac tgg t 3`) was located 47bp downstream of the stop codon of the gene. Direct sequencing of the 1446bp PCR fragments were carried out using a commercially available sequencing kit (BigDye terminator v1.1, Applied Biosystems, Foster City, USA) and the ABI 3500XL sequencer according to the manufactures instructions (Applied Biosystem).

Analysis of sequence data (entire *ppE18* gene: 1176bp) and SNP detection was performed by using SeqScape v2.6 software (Applied Biosystems). Genome sequences of *M*. *tuberculosis* H37Rv (http://tuberculist.epfl.ch/) were used as a reference sequence.

Statistical analysis was performed using GraphPad Prism v.5 (GraphPad Software Inc., USA).

## Results

To investigate the genomic diversity of the *ppE18* gene (Rv1196) coding for a protein subunit of the TB vaccine candidate M72/AS01E, direct sequencing of the entire gene (1176bp) was successfully performed in a reference collection of 71 MTBC strains which represent all major phylogenetic lineages described for the MTBC (three strains for most of the lineages, Table 1). This collection included 57 strains of human-adapted (*M*. *tuberculosis*, *M*. *africanum* and *M*. *canettii*) as well as 11 strains of animal adapted lineages (*M*. *bovis*, *M*. *microti*, *M*. *pinipedii*, *M*. *caprae*) and the ATCC strains H37Rv, *M*. *bovis* and *M*. *africanum*. All strains were previously classified into phylogenetic lineages based on spoligotyping, MIRU-VNTR and deletion typing [26]. Sequences obtained were compared to the H37Rv reference genome to identify possible variations e.g. SNPs, deletions or insertions.

In total, we found 100 sequence gene variations consisting of 96 SNPs, three insertions (24bp, 6bp, 12bp) and one deletion (3bp). This translates to 141 variable positions in the 1176bp fragment of the *ppE18* gene resulting in an average mutation density of 1.69 x 10^−3^/bp (S1 Table). According to previous findings, the majority of the 96 SNPs detected were non-synonymous (n = 68, 70.8%, S1 Table) leading to an amino acid replacement; 28 of them were synonymous (n = 28, 29.2%, S1 Table). Interestingly, transversions and transitions events were equally distributed (transversions n = 45, transitions n = 52, S1 Table), leading to this wide range of amino acid substitutions. However, 16 out of 68 non-synonymous SNPS detected are considered to be conservative mutation which might not impact the protein folding and function. Two of the insertions (6bp INS of Alanine [gcg] and Threonine [acg]; 12bp INS of Glutamine [cag], Leucine [ctg], Glycine [ggt] and Serine [tcg]) and the deletion (3bp DEL of Alanine [gcc]) could be detected at in-frame positions not interrupting the coding sequence (S1 Table and S2 Table). The 24bp INS could be defined as a frameshift mutation interrupting the coding sequence between position 341 and 342 after the start codon of the gene (S1 Table and S2 Table).

**Fig 1:**
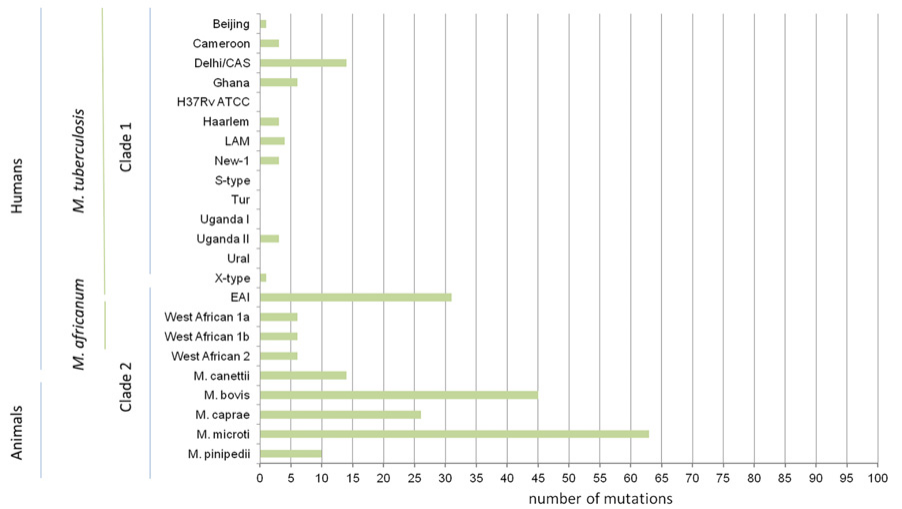
Distribution of 100 sequence variation among different MTBC lineages. ATCC: American Type Culture Collection; CAS: Central Asien; EAI: East African Indian; LAM: Latin American Mediterranean; Tur: Turkish.

Overall, the level of sequence variability differs between strains of different phylogenetic lineages (Fig 1). Animal adapted strains showed a significant higher diversity than the human pathogens e.g. all *M*. *microti* strains investigated showed 60 lineage specific and three strain specific SNPs (Fig 1 and S1 Fig [p<0.05], S2 Table). In contrast to that, the closely related *M*. *pinipedii* strains just have ten SNPs in common. However, to compare the genetic diversity between *M*. *microti* and *M*. *pinnipedii* strains, further analysis of these specific lineages with more clinical isolates have to be performed. In general, all animal adapted strains share SNP patterns confirming the deep branching of clade 1 and clade II (S2 Table, highlighted in green).

**Fig 2:**
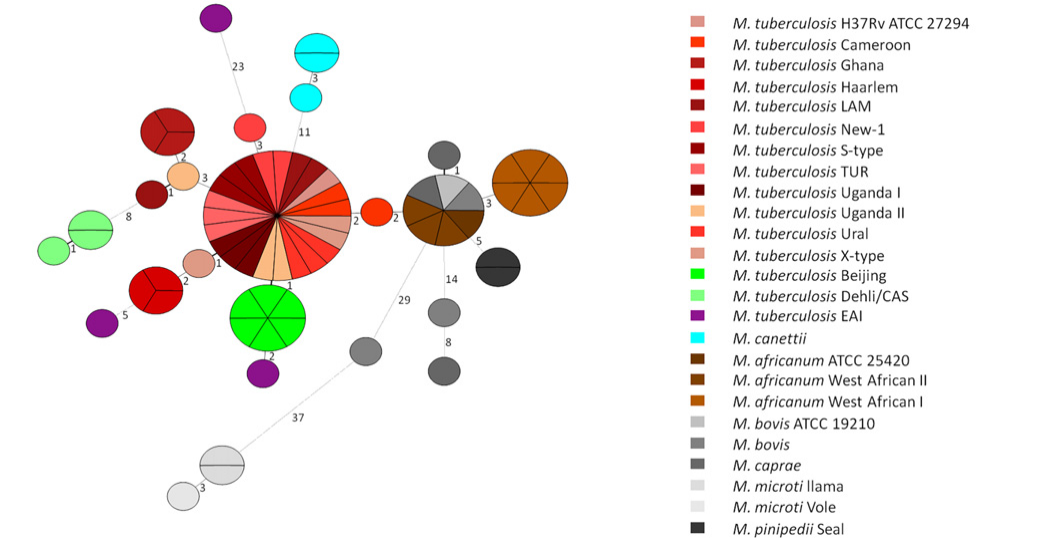
Minimum spanning tree based on 96 SNPs detected in the reference collection. Indicated numbers present SNP differences between the strains investigated. Nodes are highlighted in correspondence to the phylogenetic lineages. ATCC: American Type Culture Collection; CAS: Central Asien; EAI: East African Indian; LAM: Latin American Mediterranean; Tur: Turkish.

When clade 1 strains are considered, overall sequence variability is lower. Most of the strains investigated do not carry more than 6 SNPs in comparison to the H37Rv reference genome. Strains of four lineages (*M*. *tuberculosis* S-type, Tur, Uganda I and Ural) showed no specific mutation at all, confirming their close relationship (Fig 2). Shared SNP patterns between all human adapted strains are comparatively rare (S2 Table, highlighted in blue).

Interestingly, *M*. *africanum* and *M*. *canettii* strains, which both belong to the same phylogenetic group as the genetic divers animal adapted strains (clade 2), showed only 6 and 14 sequence variations comparable to the SNP occurrence in *M*. *tuberculosis* Dehli/CAS or Ghana strains which belong to the same phylogenetic group as the H37Rv reference strain. As *M*. *canettii* strains are considered as the precursor forms of modern *M*. *tuberculosis* strains, the low genetic diversity in this hypervariable gene is an unusual observation. Based on the evolutionary age of *M*. *canettii* strains, these strains normally showed a high amount of sequence variations in comparison to the H37Rv reference strain. In contrast to that, *M*. *tuberculosis* EAI strains showed 30 SNPs in total, whereas 26 of them were only acquired in one of three EAI strains investigated (S2 Table). Further analysis of the *ppE18* gene of more clinical isolates of this specific lineage is ongoing.

## Discussion

TB still remains one of the leading infectious diseases worldwide. The World Health Organization (WHO) declared in 2001 to eliminate TB until 2050 [27]. However, in our current situation especially considering the emergence of multidrug and extremely drug resistant strains, this global aim cannot be achieved. Therefore it is all even more important and necessary do develop an efficient vaccine interrupting ongoing infection and transmission of the disease. However, development of an effective vaccine might be challenged by the genetic diversity within the MTBC that was shown to be higher than previously assumed [12,18,28,29]. Accordingly, the analysis of sequence variability’s among MTBC genes coding for protein subunits of new vaccine candidates is urgently needed to estimate the potential to induce a protective immune response against a wide range of MTBC strains of different phylogenetic groups which occurrence has a strong geographical dependence. Here, we investigated the genetic diversity of the *ppE18* gene encoding for a subunit of the new vaccine candidate M72/AS01E. The analysis of a well-characterized reference collection of 71 MTBC strains representing 23 different phylogenetic lineages from different geographical settings provided a comprehensive insight into possible sequence variants which can be used to optimize the vaccine induced immunological response against a wide range of MTBC strains. With an average mutation density of 1.69 x 10^−3^/bp, sequence variability was extraordinary high compared to an average variability observed in recent genome studies [30]. Although 16 out of 68 SNPs detected are conservative mutations which might not directly change the folding of the PPE18 protein, the high amount of sequence variations within the entire gene can lead to significant differences in the protein structure. However, to understand the impact of the mutation density on the protein function, molecular modeling approaches as well as functional analysis of this protein is urgently needed.

PE-PPE proteins (Pro-Glu [PE] and Pro-Pro-Glu [PPE]), comprising 10% of the coding capacity of the MTBC genome [31], and are involved in several pathobiological processes [32–35]. It was shown that in contrast to the N-terminal end of the PE-PPE protein superfamily the C-terminal remains highly variable [35] which contributes to e.g. antigenic variation and helps the pathogen to escape from antimicrobial defense mechanism of the host [33]. However, in our study, sequence analysis of the *ppE18* gene revealed a quite high diversity among the entire gene whereas most of the mutations leading to an amino acid exchange. In comparison to other MTBC gene classes like housekeeping genes or genes encoding for surface molecules, the ratio of non- synonymous SNPs (nSNPs) to synonymous SNPs (sSNPs) is extremely high (nSNPs:sSNPs housekeeping 1.59; nSNPs:sSNPs surface 1.60; nSNPs:sSNPs *ppE18* 2.43) [29]. Most of the mutations with potential functional impact were detected in animal adapted strains as well as in *M*. *tuberculosis* EAI strains which might explain discrepancies to previous findings described by Mortier et al. postulating a high level of sequence conservation among MTBC strains in this particular gene [36]. However, Mortier and colleagues excluded all animal adapted strains as well as the *M*. *canettii* lineage. In contrast to human restricted strains, a higher genetic diversity within the animal adapted lineages might reflect a strong and enduring selection to escape the immune response induced by a bright range of hosts cells of different animal reservoirs [37]. Another possible explanation might be an increased rate of homologous recombination events enforced by the adaptation to different host environments in comparison to classical *M*. *tuberculosis* strains [33]. Whether the genetic diversity and immunity of host cells shape the MTBC pathogen evolution should be investigated in future studies. Furthermore, the evolutionary impact of the host-pathogen-interaction on vaccine efficacy has to be elaborated. Based on our sequence analysis, an epitope prediction study which was perfomed by McNamara et al. in 2010 [11] has to be repeated to understand the impact of the genetic diversity of the host cells as well as of different MTBC lineages on the vaccine efficacy. McNamara and colleagues predicted that the MTB72f vaccine is less effective for several DRB1 genotypes occuring especially in high burden TB countries due to limited vaccine epitope binding or to binding primarily by unconserved PPE18 epitopes [11].

Nair et al. showed that PPE18 inhibits proinflammatory cytokine production (e.g. Il12p-40) by binding to TLR2 receptors and by inducing Il-10 secretion in macrophages[38,39]. The TLR2 interacting domain is localized within the N-terminal PPE domain whereas amino acid 1–180 are essential [39]. In our study, we detected 36 mutations in this specific area, most of them leading to an amino acid exchange (S1 Table). Especially animal adapted strains show a genetic variability in this N-terminal domain which might explain the comparable low immunogenicity of the *M*. *tuberculosis* antigen in BCG vaccinated and *M bovis* infected cattles and argue for specific immune adaptation due to a wider range of host species [40]. However, if the genetic diversity observed also plays a significant role on the modulation of a protective anti-inflammatory immune response in humans has to be further investigated.

## Supporting Information

S1 Fig Box-Whisker-Plot indicate the mutation distribution among MTBC strains. Mann-Whitney test showed significant differences between human and animal adapted MTBC lineages (p<0.05).
(TIF)

S1 Table Mutations detected in the reference collection. (XLSX)

S2 Table Mutations detected in each strain of the reference collection. Shared SNP patterns are highlighted. Green: among animal adapted strains; Blue: among human pathogens; Pink: among human and animal adapted strains
(XLSX)
