# Supplementary figures and images for: High Sequence Variability of the ppE18 Gene of Clinical Mycobacterium tuberculosis Complex Strains Potentially Impacts Effectivity of Vaccine Candidate M72/AS01E
Source: PLoS One. 2016 Mar 24;11(3):e0152200. doi: 10.1371/journal.pone.0152200 (PMC4806982; doi:10.1371/journal.pone.0152200)

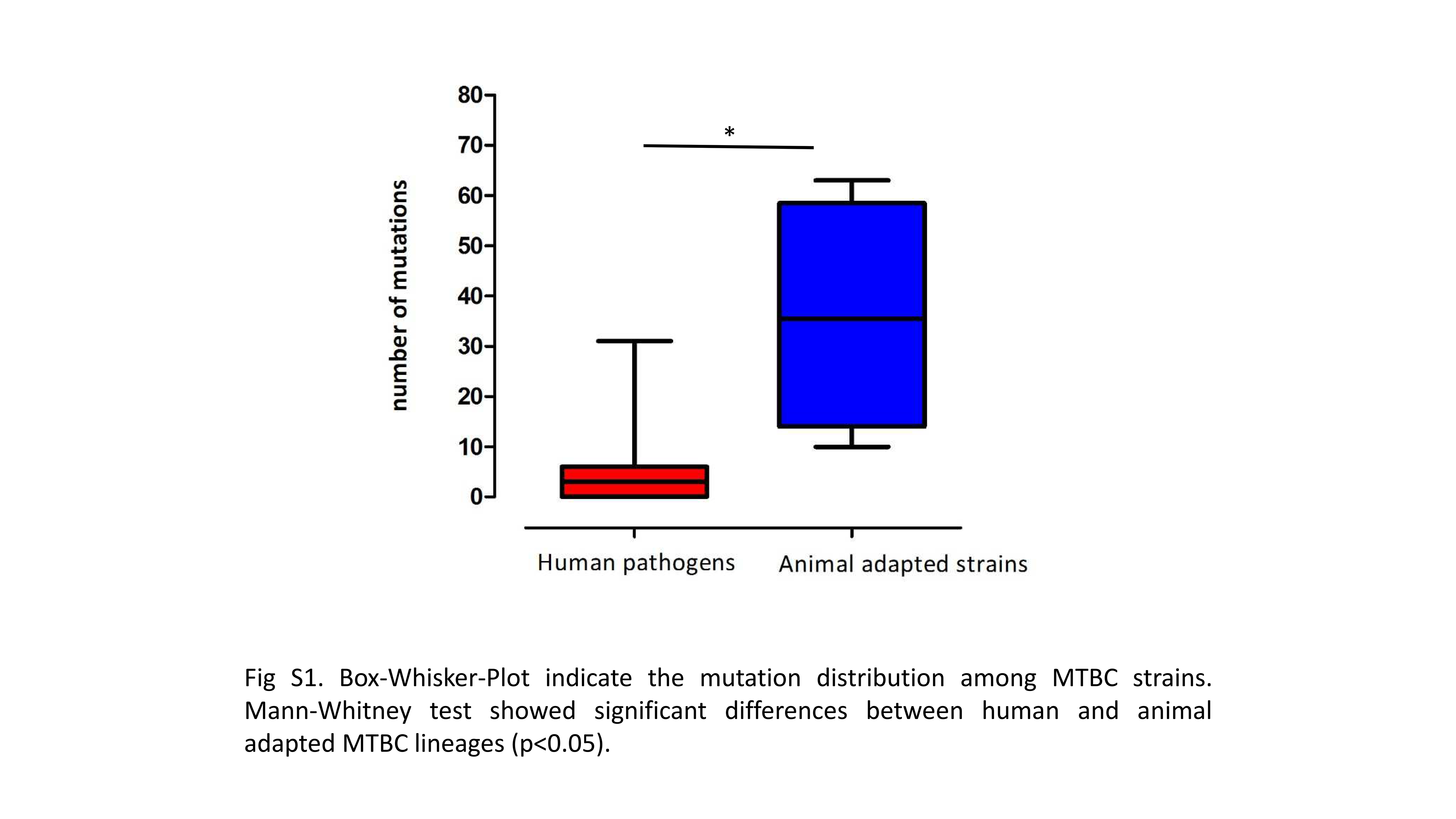

Supplement: S1 Fig — Mann-Whitney test showed significant differences between human and animal adapted MTBC lineages (p<0.05). (TIF) [file pone.0152200.s001.tif]
